# A Novel Acquired t(2;4)(q36.1;q24) with a Concurrent Submicroscopic del(4)(q23q24) in An Adult with Polycythemia Vera

**DOI:** 10.3390/cancers10070214

**Published:** 2018-06-25

**Authors:** Eigil Kjeldsen

**Affiliations:** Cancer Cytogenetic Section, HemoDiagnostic Laboratory, Department of Hematology, Aarhus University Hospital, Tage-Hansens Gade 2, DK-8000 Aarhus C, Denmark; Eigil.Kjeldsen@clin.au.dk; Tel.: +45-7846-7398; Fax: +45-7846-7399

**Keywords:** polycythemia vera, chromosomal abnormality, submicroscopic deletion del(4q), oligonucleotide array-based Comparative Genomic Hybridization (aCGH)

## Abstract

*Background*: Polycythemia vera (PV) is a clonal myeloid stem cell disease characterized by a growth-factor independent erythroid proliferation with an inherent tendency to transform into overt acute myeloid malignancy. Approximately 95% of the PV patients harbor the *JAK2*V617F mutation while less than 35% of the patients harbor cytogenetic abnormalities at the time of diagnosis. *Methods and Results:* Here we present a *JAK2*V617F positive PV patient where G-banding revealed an apparently balanced t(2;4)(q35;q21), which was confirmed by 24-color karyotyping. Oligonucleotide array-based Comparative Genomic Hybridization (aCGH) analysis revealed an interstitial 5.4 Mb large deletion at 4q23q24. Locus-specific fluorescent in situ hybridization (FISH) analyses confirmed the mono-allelic 4q deletion and that it was located on der(4)t(2;4). Additional locus-specific bacterial artificial chromosome (BAC) probes and mBanding refined the breakpoint on chromosome 2. With these methods the karyotype was revised to 46,XX,t(2;4)(q36.1;q24)[18]/46,XX[7]. *Conclusions*: This is the first report on a PV patient associated with an acquired novel t(2;4)(q36.1;q24) and a concurrent submicroscopic deletion del(4)(q23q24). The study also underscores the benefit of combined usage of FISH and oligo-based aCGH analysis in characterizing chromosomal abnormalities. The present findings provide additional clues to unravel important molecular pathways in PV to obtain the full spectrum of acquired chromosomal and genomic aberrations, which eventually may improve treatment options.

## 1. Introduction

Polycythemia vera (PV) is a clonal hematopoietic stem cell disorder classified as a *BCR/ABL1*-negative myeloproliferative disease (MPD) with a variable risk of transformation into myelodysplasia (MDS) or acute myeloid leukemia (AML) [1,2]. It is characterized by a clonal increase in red blood cells, granulocytes and platelets, with erythrocytosis being the hallmark of the disease. The major contributor to the death of patients with PV appears to be AML/MDS, but there is no generally applicable way to predict which patient is likely to acquire this fatal complication.

Cytogenetic abnormalities have been found in approximately 13–35% of patients with PV at the time of diagnosis [3,4,5,6,7,8,9,10]. The most common chromosomal abnormalities at diagnosis of PV are trisomies of chromosomes 1, 8, and 9, as well as del(20q). Their role in the pathogenesis of the disease remains largely obscure and none of them are specific to PV. Although some reports have suggested that patients with PV carrying chromosomal aberrations at the time of diagnosis have a shorter survival and increased risk of AML/MDS compared to those with a normal karyotype [11] the predictive prognostic value of chromosomal aberrations has not yet been established in PV.

A decade ago the first reports on oncogenic mutations in *JAK2* appeared [12,13,14,15,16]. It has been found that more than 95% of the PV patients harbor the common *JAK2*V617F mutation although with varying allelic burden. The mutation is not specific to PV as it is also found in other MPN’s although with lower frequency as well as in MDS/AML albeit more rarely. The *JAK2*V617F mutation is apparently not the disease-initiating event in humans, although the mutation in mice models has been found to induce a PV-like phenotype [17]. Still, no genetic defect entirely specific to PV has been identified.

Here we utilized several fluorescent in situ hybridization (FISH) applications an oligonucleotide array-based Comparative Genomic Hybridization (aCGH) analysis in combination to characterize a *JAK2*V617F positive PV patient harboring the novel acquired t(2;4)(q36.1;q24) and a concurrent interstitial microdeletion at 4q23q24.

## 2. Results

### 2.1 Clinical Description

A 64-year-old female presented with a weight loss of 2-3 kg over a period of 3 months, pruitus and intermittent night sweat. Biochemical analysis revealed a hemoglobin concentration of 13.2 mmol/L (reference interval (RI) females: 7.3–9.5) and hematocrit >0.60 (RI females: 0.35–0.46). The platelet count was 372 × 10^9^/L (RI: 165–400 × 10^9^/L), and the leucocyte count was 10.9 × 10^9^/L (RI: 3.5–10.0 × 10^9^/L). The reticulocyte count was increased at 145 × 10^9^/L (RI: 31–97 × 10^9^/L). The plasma erythropoietin was suppressed at <1.0 IU/L (RI: 5–30 IU/L). A bone marrow biopsy displayed features characteristic of PV with trilineage hyperplasia. The spleen was not enlarged upon clinical examination. The patient had a *JAK2*V617F mutation with an allelic burden of 71% and FISH excluded *BCR-ABL1* fusion gene. The patient was treated with phlebotomy, acetylsalicylic acid and required cytoreductive therapy with hydroxyurea or pegylated interferon alfa-2a due to intermittent thrombocytosis and leucocytosis during disease course (Supplementary Figure S1). The total follow-up time was 64 months and she is now in continuous treatment with phlebotomy, hydroxyurea and acetylsalicylic acid.

### 2.2 Cytogenetic Analyses 

**Figure 1:**
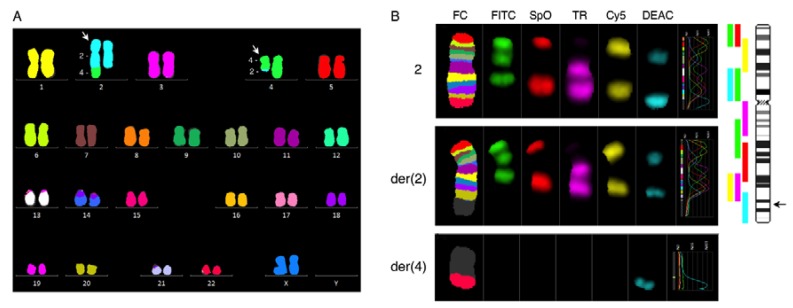
Multi-color Fluorescence in Situ Hybridization (FISH). (**A**) 24-color karyotyping revealed the translocation t(2;4). White arrows indicate the derivative chromosomes. (**B**) mBanding analysis of chromosomes 2. The single-color gallery tool in ISIS software shows assigned false colors (FC) and individual color schemes of labeled chromosomes arranged in their capture sequence FITC (fluorescein isothiocyanate), SpO (spectrum orange), TR (Texas red), Cy5 (cyanine), DEAC (7-diethylaminocoumarin-3-carboxylic acid, succinimidyl ester). Upper row shows the normal chromosome 2, middle row shows the der(2)t(2;4) and lower row show the der(4)t(2;4) from the patient’s karyotype. The right-hand side shows a schematic representation of the localization of the different multicolor probes of XCyte 2 relative to the ideogram of chromosome 2 together with breakpoint marked by the arrow.

The karyotype, examined by G-banding analysis combined with 24-color karyotyping and mBanding analysis with chromosome 2 probes, was pseudodiploid harboring an apparently balanced reciprocal translocation between chromosomes 2 and 4 described as 46,XX,t(2;4)(q35;q21)[18]/46,XX[7] (Figure 1). To establish whether this translocation was constitutional or belonged to the malignant clone a peripheral blood sample was requested. G-banding after phytohaemagglutinin (PHA)-stimulated culturing showed a normal female karyotype 46,XX[25] (data not shown) indicating that the aberrant t(2;4) belongs to the malignant clone.

### 2.3 Oligo-Based Array Comparative Genomic Hybridization (CGH) Analysis 

**Figure 2:**
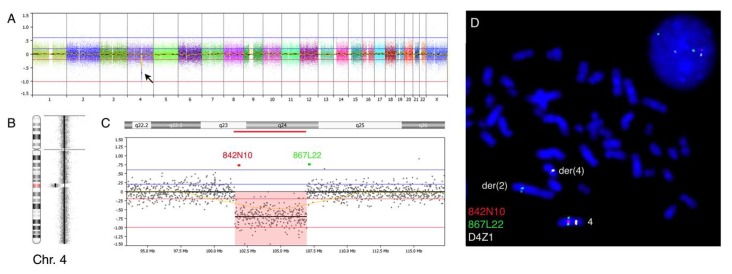
Oligo-based array Comparative Genomic Hybridization (oaCGH) analysis and FISH validation. (**A**) Whole genome view of the bone marrow sample showing a submicroscopic deletion at chromosome 4 indicated by the arrow. Horizontal blue lines indicate log_2_ ratios +0.24 and +0.60 and red lines indicate log_2_ ratios −0.24 and −1.0. The X-axis at the bottom indicates chromosomal position. (**B**) Chromosome view of chromosome 4 with deletion at 4q23-q24 indicated by an arrow and ideogram of chromosome 4 to the left. (**C**) A zoom view of the deleted region as indicated by red shade corresponding to the deletion’s maximal chromosomal position. The red and green bars indicate the position of FISH probes used for validation. (**D**) FISH using BAC (Bacterial Artificial Chromosomes) probes RP11-842N10 (red) and RP11-867L22 (green) at 4q23 and 4q24, respectively, and centromeric probe D4Z1 (aqua) confirms the interstitial mono-allelic deletion and translocation in nuclei and metaphases from the patient.

High-resolution oligo-based aCGH (oaCGH) analysis revealed an approximately 5.4 Mb large deletion at chromosome 4 band region q23 to q24 (Figure 2A,B). The minimal region of deletion encompassed the following probes A_16_P36844152 to A_16_P36856072, mapping from 101,572,440 bp to 106,955,633 bp and the maximal region of deletion encompassed probes A_16_P368440787 to A_16_P16801426 mapping from 101,550,452 bp to 106,975,209 bp. The deleted region affected 40 RefSeq genes including *TET2* and *CXXC4* (Table 1). It is to be noted that the aCGH analysis did not disclose any further copy number alterations, neither on chromosome 2.

### 2.4 Validation by FISH Analyses

To validate the above aCGH findings we performed FISH analysis using the bacterial artificial chromosomes (BAC)-based probes RP11-842N10 and RP11-867L22 together with centromeric probe D4Z1 (Figure 2C,D). This analysis confirmed the mono-allelic nature of the deletion in both metaphases and interphase nuclei and that the deletion involved the same chromosome 4 derivative being involved in the t(2;4). After counting of 200 nuclei we found that 150 nuclei exhibited a 2G1R2A pattern, indicating that 75% of the cells carried the deletion in the diagnostic sample. Fluorescence In Situ Hybridization on PHA-stimulated cultured white blood cells from peripheral blood with the probes RP11-842N10, RP11-867L22, and D4Z1 exhibited a normal signal pattern 2G2R2A in 100% of interphase nuclei and on metaphases demonstrating that the observed abnormalities are acquired and belong to the abnormal hematopoietic cells.

### 2.5 Breakpoint Mapping by FISH Analyses

**Figure 3:**
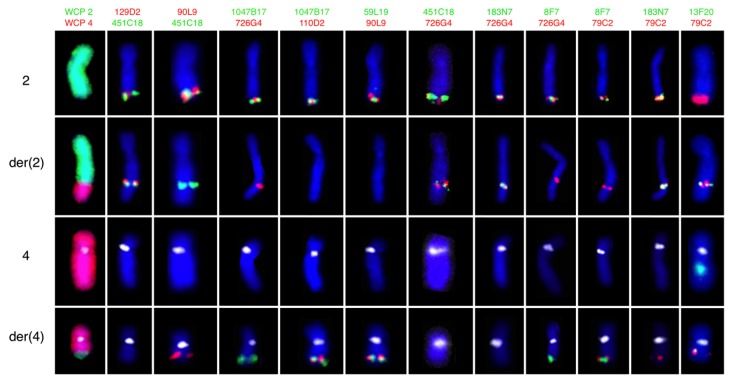
Break point mapping with FISH probes. Partial karyograms of normal chromosome 2, der(2)t(2;4), normal chromosome 4, and der(4)t(2;4) arranged from top to bottom showing FISH results after hybridization using respective dual color probe sets as indicated at the top. WCP indicates whole chromosome painting, and chromosome 4 is indicated by the centromeric D4Z1 probe (aqua).

To establish the breakpoint region on chromosomes 2 and 4 more precisely we used a panel of BAC-based probes in different combinations (Table 2 and Figure 3). From these FISH analyses we were able to determine that the breakpoint region on chromosome 2 is located within the BAC-probe RP11-79C2 and on chromosome 4 within the BAC-probe RP11-13F20. 

**Figure 4:**
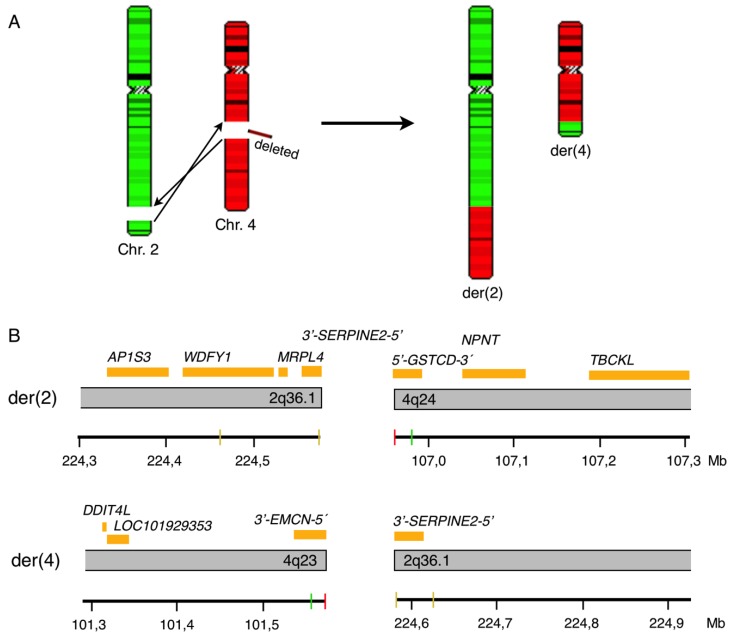
Model of chromosomal rearrangement. (**A**) Schematic representation of the translocation between chromosomes 2 and 4 with breakage points indicated by arrows together with the deleted region followed by reciprocal joining. (**B**) Schematic representation of genes (light brown boxes) mapping in correspondence to the breakpoint regions. Upper panel shows the joined regions of 2q36.1 and 4q24 and the lower panel shows the joined regions 4q23 and 2q36.1. The deleted chromosomal fragment del(4)(q23q24) is omitted and the genes located in this region are listed in Table 2. The axis at the bottom of each panel indicates the chromosomal position of the involved regions. The resolution of the aCGH analysis is limited to the kilobase pair level and the density of the oligo probes differ according to chromosomal regions with the highest density at known cancer genes. Vertical red and green bars indicate the relative genomic position of deleted (red) and not deleted (green) oligonucleotide probes in the aCGH analysis. Vertical yellow bars indicate the position of BAC clone RP11-79C2 being involved in the translocation.

Taken together, we have shown that an approximately 5.4 Mb large chromosomal segment encompassing the bands 4q23q24 is deleted and that this event is accompanied by a reciprocal translocation of the telomeric 4q24-qter segment with the 2q36.1-qter segment as summarized in Figure 4A. The final karyotype resulting from conventional cytogenetics, FISH and aCGH investigations, according to ISCN 2013, of bone marrow at diagnosis is: 46,XX,der(2)t(2;4)(q36.1;q24),der(4)del(4)(q23q24)t(2;4)(q36.1;q23)[18]/46,XX[7].nuc ish(RP11-842N10x1,RP11-867L22x2,D4Z1x2)[150/200].arr[hg18] 4q23q24(101,561,446-106,965,421)x1.

In silico analysis of the involved regions suggested that the 5′-part of *EMCN* gene (spanning exons 1 to 5) at 4q23 and the 5′-part of *GSTCD* gene (spanning exons 1 to 5) at 4q24 were deleted (Figure 4B). The fusion of the chromosomal regions 4q23 and 2q36.1 on der(4)t(2;4) could theoretically form the fusion gene *SERPINE*-*EMCN*. The fusion on der(2)t(2;4) involved the 5′-part of the *GSTCD* gene on 4q24, but it could not be determined which of the *WDFY1*, *MRPL4* or *SERPINE* genes on 2q36.1 that might be involved in the translocation event due to lack of genomic resolution. Unfortunately, we could not perform gene expression or RNA sequencing analyses to establish possible presence of a fusion gene or altered expression of involved genes due to lack of additional sample material.

## 3. Discussion

Polycythemia vera has an inherent tendency to transform into myelofibrosis (MF), MDS or AML [18]. The cumulative incidence of post-PV MF evolution is 5–14% at 15 years [19,20,21] and for post-PV MDS/AML the estimated transformation rates are 2.3% at 10 years and remains <10% at 20 years [22,23]. The survival rates of PV shorten after transformation to either MF or MDS/AML. Factors influencing these survival rates include: age, leukocytosis, abnormal karyotype, splenomegaly, bone marrow reticulin grade, and *JAK2*V617F mutant allele burden [19,23,24,25,26,27,28]. It is evident that the process of transformation in PV is complex and at present it is not possible to accurately predict which PV patients that will transform or not. 

Cytogenetic abnormalities may play a role in the transformation process as the frequency of chromosomal aberrations in PV is approximately 20% at diagnosis but much higher among patients transforming to AML [7,8,9,10]. Trisomy 8 and 9, and abnormalities of chromosome 1, 20q-, 13q-, 11q-, 3p-, and dup13 have most frequently been observed, especially in older patients >60 years of age [4,7]. A minority of PV patients have been shown to have acquired various translocations at diagnosis including t(2;11)(p21;q23) [29], t(16;20)(q22;p13) [30], and der(18)t(9;18)(p13;p11) or der(9;18)(p10;q10) [31]. However, the role of these different types of cytogenetic abnormalities in transformation of PV remains obscure, and cytogenetic abnormalities have not been shown to carry prognostic relevance [5].

In this study, we found a t(2;4)(q36.1;q24) in a female with *JAK2*V617F-positive PV at diagnosis with a follow-up period of 5 years where her disease has not transformed to MF or MDS/AML. The identified t(2;4) translocation is to the best of our knowledge novel as searches in Mitelman [32] and literature databases revealed no additional cases. In addition, a search in our local registry with more than 20,000 entries of different hematological malignancies since 2001 was also without additional cases. By aCGH analysis we detected an additional concurrent submicroscopic 5.4 Mb large deletion at 4q23q24, and it was confirmed by FISH analyses that the deletion was located on the der(4)t(2;4) at the 4q24 translocation breakpoint. Both of these chromosomal abnormalities were acquired because cytogenetic analyses of surrogate germ-line cells were without the detected aberrations.

Chromosomal aberrations involving chromosome band 2q36 have been reported in 15 MDS or AML cases [32]. The reported aberrations include: t(1;2)(q21-q22;q36) [33,34,35], t(X;2)(p22;q36) [36], t(2;17)(q36;q11) [37,38], and del(2)(q23-q35q36) [39,40,41]. In the present PV patient, we performed chromosome walking utilizing several different FISH probes and mapped the translocations breakpoint to be within the BAC-clone RP11-79C2 at 2q36.1 (genomic pos. 224,460,665–224,627,199; hg18). There are three genes located within this BAC-clone: *WDFY1*, *MRPL44* and *SERPINE2*. In silico analysis revealed that *SERPINE2* theoretically could form a fusion gene with the *EMCN* at 4q23 (Figure 4). The *SERPINE2* gene, expressing a serine proteinase inhibitor clade E member 2, was recently shown to be a potential biomarker for tumor diagnosis and prognosis in a variety of solid tumors [42]. Although the *EMCN* gene previously has been shown to be overexpressed in acute leukemia [43] further studies are needed to clarify the potential role, if any, of the *SERPINE2* and *EMCN* genes in the pathogenesis of the present patient.

Chromosome band 4q24 is a more common chromosomal breakpoint region in myeloid malignancies as 34 cases have been reported in MDS or AML [32]. The most frequent aberrations include: t(1;4)(p32;q24) [44], t(1;4)(p35;q24) [45], t(4;12)(q24;q12-q22) [46,47,48], t(4;17)(q24;q25) [47,48], t(X;4)(q22;q24) [49], and del(4)(q21-q28) [50,51,52,53,54,55,56]. In a study of four female AML or MDS patients the 4q24 band was involved in three apparently balanced translocations between 4q and a variable partner chromosome, including t(3;4)(q26;q24), t(4;5)(q24;p16), and t(4;7)(q24;q21), and one patient with a localized del(4)(q24q25) [57]. Unexpectedly, the authors detected a concurrent submicroscopic deletion at 4q24 (localized between genomic positions 105,800,776 to 106,847,377; hg18) in all three patients harboring the apparently balanced translocations by metaphase FISH analyses using near-contiguous BAC-based probes. Interestingly, in our present patient we also detected a concurrent submicroscopic deletion, del(4)(q23q24), in addition to the t(2;4)(q36.1;q24). Taken together, it seems that the 4q24 chromosomal region may be directly or indirectly associated with pathogenesis in a subset of hematological disorders.

In our patient aCGH analysis defined the concurrent deletion to be 5.4 Mb large encompassing 40 RefSeq genes, including *TET2* and *CXXC4*. *TET2* belongs to a group of ten-eleven-translocation (TET) proteins, Fe(II)- and α-ketoglutarate-dependent oxygenases, that modify 5-methylcytosine (5-mC) to e.g., 5-hydroxymethylcytosine (5-hmC) [58,59], which seems to be an important step in the active demethylation of DNA [60]. Loss of *TET2* function is associated with a continuous enlargement of the hematopoietic stem cell compartment leading to myeloproliferation by a mechanism of increased hematopoietic stem cell self-renewal and myeloid transformation as indicated by studies in mice [61,62]. *TET2* mutations have been described in a wide range of myeloid malignancies with a mutation frequency of 7 to 13% [63,64]. These mutations are typically small deletions, insertions, or nonsense mutations that are expected to induce loss-of-function in the protein. The catalytic activity of TET2 might be impaired by missense mutations affecting conserved amino acids in TET2, which can result in lower global 5-hmC levels in TET2-mutated patients compared with wild-type TET2 [65].

The *CXXC4* gene is a negative regulator of Wnt signaling pathway and also regulates the *TET2* expression, where co-expression of *CXXC4* and *TET2* resulted in a decrease in levels of 5-hmC [66]. The homeostatic self-renewal of stem cells in adult tissues is regulated by the Wnt signaling pathway [67], and its constitutive activation contributes to cancer development and progression [68,69,70]. Other genes involved in regulation of Wnt pathways include the *ASXL1, ASXL2, UTX, CXXC4, CXXC5, TET2*, and *TET3* genes as indicated by MDS patients harboring mutations in these genes [71]. The *CXXC4* gene has been associated with development of renal carcinoma [72], colonic villous adenoma [73], and gastric cancer [74].

Our study patient had, in addition to the t(2;4) and del(4)(q24q24), a high allelic burden of *JAK2*V617F mutation (equivalent to 71%) which was similar the number of cells harboring the 4q24 deletion. This finding is in line with observations that the most common co-occurring classes of mutations in MPNs are signaling mutations (e.g., *JAK2*V617F) and mutations in genes involved in epigenetic regulation (e.g., *TET2*) [75]. Progression in PV patients may result from co-occurring mutations in *JAK2*V617V and *TET2* because loss of *TET2* might drive clonal dominance in hematopoietic stem cells and that *JAK2*V617V expression might cause expansion of precursor cell populations [75]. Furthermore, it was observed that treatment of PV patients carrying both *TET2* and *JAK2* mutations experienced reduction in the *JAK2* mutant clones without significant eradication of the *TET2* mutant clone [76]. This was also observed in PV patients during PEG-IFN-α-2a therapy where the *TET2* mutant clones persist despite eradication of *JAK2*V617F clones [75]. In some patients pegylated interferon alfa-2a has the ability to induce complete bone marrow responses [77]. Our patient was initially treated with phlebotomy, cytoreductive (hydroxyurea) and anti-thrombotic therapy but due to persistent pruritus and high platelet counts cytoreductive treatment was changed to pegylated interferon alfa-2a for a period of approximately 14 months (Supplemental Figure S1). Unfortunately, we do not have any molecular follow-up data on allelic burden of *JAK2*- or *TET2*-mutations to document potential changes in mutation burdens of these mutations but she responded clinical well. Another limitation of this study is that the follow-up of the present patient is relatively short, a little more than 5 years, as it is known that PV may transform after up to 20 years.

Only a few studies have used aCGH or single nucleotide polymorphism (SNP) analysis in characterizing PV genomic aberrations. In a study of 26 PV patients high density oligo-based aCGH analysis detected copy number alterations in 35% of the patients at diagnosis [78]. Another study of 14 PV patients concluded that microdeletions and microduplications do not have an essential role in the development of PV as detected by high-density oligo-based aCGH analysis [79]. A study using high-resolution SNP microarrays revealed a common uniparental disomy (UPD) of chromosome 9p or gain of 9p in addition to other copy number aberrations in a small cohort of post-PV MF patients with elevated *JAK2*V617F mutation burden [80]. Our present study together with these previous studies underscores the value of using aCGH or SNP analysis in characterizing genomic alterations in PV at least in subsets of patients with e.g., translocations involving chromosome band 4q24.4.

## 4. Materials and Methods

### 4.1 Cytogenetic Analysis

Unstimulated overnight cultures of bone marrow sample from the patient were examined according to our standard laboratory protocols. Phytohemagglutinin (PHA)-stimulated culture from a peripheral blood sample was established at a later time point to examine whether identified chromosomal abnormalities were acquired or congenital. Chromosome preparations were treated and stained by Giemsa-banding. Karyotypes were described according to *An International System for Human Cytogenetic Nomenclature* (ISCN, 2013) [81]. Written informed consent was obtained from the patient.

### 4.2 FISH Analysis

To characterize the chromosome rearrangement multicolor FISH were done on chromosome preparations from bone marrow according to manufacturer’s instructions using the following human XCyting multicolor FISH probes (MetaSystems, Altlussheim, Germany): (1) 24-color karyotyping was done with the 24XCyte kit consisting of 24 different chromosome painting probes; and (2) mBanding with XCyting probes for chromosome 2 consisting of a series of partial chromosome paint probes for sequential partially overlapping chromosome regions of a single chromosome. Each of the XCyte probes was labeled with one of five fluorochromes or a unique combination thereof (combinatorial labeling). Metaphases were counterstained with 4′,6-diamidino-2-phenylindole (DAPI). Image capture was done with an automated Zeiss Axio Imager.Z2 equipped with a couple-charged device (CCD)-camera (CoolCube1, MetaSystems, Altlussheim, Germany) and appropriate filters (MetaSystems, Altlussheim, Germany). Karyotyping was done using the 24-color mFISH upgrade package, ISIS, including mBanding.

Locus-specific directly fluorescent-labeled BAC probes (Empire Genomics, Buffalo, NY, USA) on chromosomes 2 and 4 were used for validation of identified microdeletion by oaCGH analysis and break point mapping together with SE4 (D4Z1) (Kreatech, Amsterdam, The Netherlands). To estimate the number of abnormal cells 200 interphase nuclei was evaluated by two independent observers. All locus-specific analyses were done according to manufacturer’s instructions. To identify the common dual fusion probe *BCR-ABL1* (Abbott Molecular, Wiesbaden, Germany).

### 4.3 oaCGH Analysis

The CytoChip Cancer 4 × 180 K v2.0 (BlueGnome, Cambridge, UK) encompassing a 20 kb backbone with highest concentration of probes at 670 cancer genes was used for oaCGH analysis according to manufacturer’s instructions as described in [82]. DNA purified from bone marrow cells was used together with pooled female genomic DNA as reference. After hybridization, washing and drying, the oligo array was scanned at 2.5 µm with GenePix 4400A microarray scanner. Initial analysis and normalization was done with BlueFuseMulti v2.6. For analysis and visualization normalized log2 probe signal values were imported into Nexus Copy Number software v. 6.1 (BioDiscovery, CA, USA) and segmented using FASST2 segmentation algorithm with a minimum of 3 probes/segment. Regions of gain or loss contained within copy number variable regions (CNVs) were discarded. Reference genome was NCBI build 36.1 (hg18). The UCSC database (http://genome.ucsc.edu) was used for bioinformatics analysis.

### 4.4 JAK2 Mutation Analysis

DNA extracted from peripheral blood leukocytes after red blood cell lysis was used for *JAK2*V617F mutation analysis, which was done by allele-specific polymerase chain reaction as described [12].

## 5. Conclusions

In summary, we identified a novel apparently balanced t(2;4)(q36.1;q24) with a concurrent cryptic del(4)(q23q24) in a *JAK2*V617F positive PV patient. The submicroscopic deletion was detected by aCGH analysis and found to be 5.4 Mb in size encompassing 40 RefSeq genes, including *TET2* and *CXXC4*. FISH analysis confirmed that the interstitial submicroscopic mono-allelic deletion at 4q was on der(4)t(2;4).

Our findings agree with observations from mice model systems where concomitant *JAK2*V617F expression and *TET2* loss promote accelerated myeloproliferation but no overt fibrotic or leukemic transformation. It is, therefore, important to identify PV patients that are positive for both *TET2*- and *JAK2*-mutations to offer optimal treatment options. To identify this group of *JAK2*V617F positive PV patients harboring concomitant *TET2* loss we suggest including either locus-specific FISH analysis covering the *TET2* locus or oaCGH analysis to detect copy number changes in chromosome band 4q24.

The observed del(4)(q23q24) in our patient also encompassed the *CXXC4* gene which is a known regulator of *TET2* expression, but the impact of this observation must await further studies.

The present findings provide additional clues to unravel important molecular pathways in PV to obtain the full spectrum of acquired chromosomal and genomic aberrations. As more cases become characterized at the molecular level this may eventually improve on treatment options.

## Figures and Tables

**Table 1:** Genes located in the deleted region on chromosome 4.

Gene Symbol	Chromosome	Start	End	Length (bp)
EMCN	chr4	101,535,520	101,658,273	122,754
LINC01216	chr4	101,800,458	101,815,293	14,836
DKFZp761L0516	chr4	102,163,609	102,487,057	323,449
PPP3CA	chr4	102,163,609	102,487,651	324,043
MIR8066	chr4	102,380,974	102,381,052	79
FLJ20021	chr4	102,487,956	102,489,063	1108
AK000028	chr4	102,487,959	102,489,062	1104
BANK1	chr4	102,560,140	103,214,992	654,853
SLC39A8	chr4	103,391,220	103,485,678	94,459
NFKB1	chr4	103,641,517	103,757,507	115,991
MANBA	chr4	103,771,690	103,901,196	129,507
LOC102723704	chr4	103,917,265	103,939,844	22,580
CR618043	chr4	103,934,626	103,936,350	1725
UBE2D3	chr4	103,934,619	104,009,491	74,873
AK093356	chr4	103,968,428	103,984,352	15,925
CISD2	chr4	104,009,575	104,033,412	23,838
NHEDC1	chr4	104,025,643	104,160,325	134,683
SLC9B1	chr4	104,025,641	104,160,345	134,705
NHEDC2	chr4	104,160,837	104,217,379	56,543
SLC9B2	chr4	104,166,096	104,217,977	51,882
BDH2	chr4	104,218,230	104,240,473	22,244
UNQ6308	chr4	104,218,230	104,240,473	22,244
CENPE variant protein	chr4	104,278,956	104,281,020	2065
CENPE	chr4	104,246,411	104,339,015	92,605
LOC101929448	chr4	104,565,647	104,580,334	14,688
TACR3	chr4	104,730,073	104,860,422	130,350
AK093871	chr4	105,609,014	105,612,315	3302
CXXC4	chr4	105,608,911	105,635,507	26,597
AK094561	chr4	105,631,570	105,816,794	185,225
LOC101929468	chr4	105,631,570	105,838,198	206,629
TET2	chr4	106,286,480	106,420,409	133,930
PPA2	chr4	106,509,682	106,614,676	104,995
BC008246	chr4	106,612,473	106,614,625	2153
EEF1AL7	chr4	106,625,311	106,626,956	1646
ARHGEF38-IT1	chr4	106,702,196	106,710,841	8646
FLJ20184	chr4	106,693,225	106,772,286	79,062
ARHGEF38	chr4	106,693,225	106,821,519	128,295
AK125951	chr4	106,799,802	106,821,519	21,718
INTS12	chr4	106,823,233	106,849,330	26,098
GSTCD	chr4	106,849,389	106,988,331	138,943

Bp: base pairs.

**Table 2:** BAC-based FISH probes used to characterize the break points of the t(2;4)(q22;q24).

Cytoband	Position [hg18]	Probe	Result on Derivative Chromosomes
4q23	101,510,593–101,671,984	RP11-13F20	Partially deleted on der(4), and fused with RP11-79C2
4q23	101,560,500–101,762,033	RP11-842N10	Deleted on der(4)
4q24	107,113,145–107,319,249	RP11-867L22	On der(2)
4p10q10		D4Z1	On der(4)
2q35	218,520,847–218,639,368	RP11-129D2	On der(2)
2q36.1	221,626,882–221,799,537	RP11-451C18	On der(2)
2q36.1	224,095,528–224,291,965	RP11-726G4	On der(2)
2q36.1	224,301,090–224,460,665	RP11-183N7	On der(2)
2q36.1	224,460,665–224,627,199	RP11-79C2	The probe is unevenly split between der(2) and der(4) by 75% and 25% signal intensity, respectively. It is fused with RP11-13F20
2q36.1	224,625,674–224,809,242	RP11-8F7	On der(4)
2q36.2	225,077,009–225,253,855	RP11-110D2	On der(4)
2q36.2	225,527,112–225,706,569	RP11-1047B17	On der(4)
2q36.3	226,764,027–226,939,580	RP11-59L19	On der(4)
2q36.3	228,283,721–228,440,226	RP11-90L9	On der(4)

## Supplementary Materials

The following are available online at http://www.mdpi.com/2072-6694/10/7/214/s1, Figure S1: Longitudinal biochemical analyses and treatment overview.

## References

[1] Swerdlow S., Campo E., Harris N.L., Jaffe E.S., Pileri S.A., Stein H., Thiele J., Vardiman J.W., The International Agency for Research on Cancer (2008). WHO Classification of Tumours of Haematopoietic and Lymphoid Tissue.

[2] Tefferi A., Vannucchi A.M., Barbui T. (2018). Polycythemia vera treatment algorithm 2018. Blood Cancer J..

[3] Bacher U., Haferlach T., Kern W., Hiddemann W., Schnittger S., Schoch C. (2005). Conventional cytogenetics of myeloproliferative diseases other than CML contribute valid information. Ann. Hematol..

[4] Diez-Martin J.L., Graham D.L., Petitt R.M., Dewald G.W. (1991). Chromosome studies in 104 patients with polycythemia vera. Mayo Clin. Proc..

[5] Gangat N., Strand J., Lasho T.L., Finke C.M., Knudson R.A., Pardanani A., Li C.Y., Ketterling R.P., Tefferi A. (2008). Cytogenetic studies at diagnosis in polycythemia vera: Clinical and JAK2V617F allele burden correlates. Eur. J. Haematol..

[6] Lawler S.D. (1980). Cytogenetic studies in Philadelphia chromosome-negative myeloproliferative disorders, particularly polycythaemia rubra vera. Clin. Haematol..

[7] Rege-Cambrin G., Mecucci C., Tricot G., Michaux J.L., Louwagie A., van Hove W., Francart H., van den Berghe H. (1987). A chromosomal profile of polycythemia vera. Cancer Genet. Cytogenet..

[8] Testa J.R., Kanofsky J.R., Rowley J.D., Baron J.M., Vardiman J.W. (1981). Karyotypic patterns and their clinical significance in polycythemia vera. Am. J. Hematol..

[9] Swolin B., Weinfeld A., Westin J. (1988). A prospective long-term cytogenetic study in polycythemia vera in relation to treatment and clinical course. Blood.

[10] Sever M., Quintas-Cardama A., Pierce S., Zhou L., Kantarjian H., Verstovsek S. (2013). Significance of cytogenetic abnormalities in patients with polycythemia vera. Leuk. Lymphoma.

[11] Andrieux J.L., Demory J.L. (2005). Karyotype and molecular cytogenetic studies in polycythemia vera. Curr Hematol Rep.

[12] Baxter E.J., Scott L.M., Campbell P.J., East C., Fourouclas N., Swanton S., Vassiliou G.S., Bench A.J., Boyd E.M., Curtin N. (2005). Cancer Genome: Acquired mutation of the tyrosine kinase JAK2 in human myeloproliferative disorders. Lancet.

[13] James C., Ugo V., Le Couedic J.P., Staerk J., Delhommeau F., Lacout C., Garcon L., Raslova H., Berger R., Bennaceur-Griscelli A. (2005). unique clonal JAK2 mutation leading to constitutive signalling causes polycythaemia vera. Nature.

[14] Kralovics R., Teo S.S., Buser A.S., Brutsche M., Tiedt R., Tichelli A., Passamonti F., Pietra D., Cazzola M., Skoda R.C. (2005). Altered gene expression in myeloproliferative disorders correlates with activation of signaling by the V617F mutation of Jak2. Blood.

[15] Kralovics R., Passamonti F., Buser A.S., Teo S.S., Tiedt R., Passweg J.R., Tichelli A., Cazzola M., Skoda R.C. (2005). A gain-of-function mutation of JAK2 in myeloproliferative disorders. N. Engl. J. Med..

[16] Levine R.L., Wadleigh M., Cools J., Ebert B.L., Wernig G., Huntly B.J., Boggon T.J., Wlodarska I., Clark J.J., Moore S. (2005). Activating mutation in the tyrosine kinase JAK2 in polycythemia vera, essential thrombocythemia, and myeloid metaplasia with myelofibrosis. Cancer Cell.

[17] Tefferi A. (2010). Novel mutations and their functional and clinical relevance in myeloproliferative neoplasms: JAK2, MPL, TET2, ASXL1, CBL, IDH and IKZF1. Leukemia.

[18] Cerquozzi S., Tefferi A. (2015). Blast transformation and fibrotic progression in polycythemia vera and essential thrombocythemia: A literature review of incidence and risk factors. Blood Cancer J..

[19] Kiladjian J.J., Gardin C., Renoux M., Bruno F., Bernard J.F. (2003). Long-term outcomes of polycythemia vera patients treated with pipobroman as initial therapy. Hematol. J..

[20] Passamonti F., Rumi E., Pungolino E., Malabarba L., Bertazzoni P., Valentini M., Orlandi E., Arcaini L., Brusamolino E., Pascutto C. (2004). Life expectancy and prognostic factors for survival in patients with polycythemia vera and essential thrombocythemia. Am. J. Med..

[21] Bonicelli G., Abdulkarim K., Mounier M., Johansson P., Rossi C., Jooste V., Andreasson B., Maynadie M., Girodon F. (2013). Leucocytosis and thrombosis at diagnosis are associated with poor survival in polycythaemia vera: A population-based study of 327 patients. Br. J. Haematol..

[22] Tefferi A., Guglielmelli P., Larson D.R., Finke C., Wassie E.A., Pieri L., Gangat N., Fjerza R., Belachew A.A., Lasho T.L. (2014). Long-term survival and blast transformation in molecularly annotated essential thrombocythemia, polycythemia vera, and myelofibrosis. Blood.

[23] Tefferi A., Rumi E., Finazzi G., Gisslinger H., Vannucchi A.M., Rodeghiero F., Randi M.L., Vaidya R., Cazzola M., Rambaldi A. (2013). Survival and prognosis among 1545 patients with contemporary polycythemia vera: An international study. Leukemia.

[24] Finazzi G., Caruso V., Marchioli R., Capnist G., Chisesi T., Finelli C., Gugliotta L., Landolfi R., Kutti J., Gisslinger H. (2005). Acute leukemia in polycythemia vera: An analysis of 1638 patients enrolled in a prospective observational study. Blood.

[25] Marchioli R., Finazzi G., Landolfi R., Kutti J., Gisslinger H., Patrono C., Marilus R., Villegas A., Tognoni G., Barbui T. (2005). Vascular and neoplastic risk in a large cohort of patients with polycythemia vera. J. Clin. Oncol..

[26] Gangat N., Strand J., Li C.Y., Wu W., Pardanani A., Tefferi A. (2007). Leucocytosis in polycythaemia vera predicts both inferior survival and leukaemic transformation. Br. J. Haematol..

[27] Abdulkarim K., Ridell B., Johansson P., Kutti J., Safai-Kutti S., Andreasson B. (2011). The impact of peripheral blood values and bone marrow findings on prognosis for patients with essential thrombocythemia and polycythemia vera. Eur. J. Haematol..

[28] Passamont F., Rumi E., Pietra D., Elena C., Boveri E., Arcaini L., Roncoroni E., Astori C., Merli M., Boggi S. (2010). A prospective study of 338 patients with polycythemia vera: The impact of JAK2 (V617F) allele burden and leukocytosis on fibrotic or leukemic disease transformation and vascular complications. Leukemia.

[29] Acar K., Sucak G.T., Yagci M., Tunca Y., Haznedar R. (2006). Translocation (2;11)(p21;q23) in a patient with polycythemia vera: A novel clonal chromosome abnormality. Am. J. Hematol..

[30] Daibata M., Taguchi T., Taguchi H. (2002). A novel t(16;20)(q22;p13) in polycythemia vera. Cancer Genet. Cytogenet..

[31] Larsen T.S., Hasselbalch H.C., Pallisgaard N., Kerndrup G.B. (2007). A der(18)t(9;18)(p13;p11) and a der(9;18)(p10;q10) in polycythemia vera associated with a hyperproliferative phenotype in transformation to postpolycythemic myelofibrosis. Cancer Genet. Cytogenet..

[32] Mitelman F., Johansson B., Mertens F.E. Mitelman Database of Chromosome Aberrations and Gene Fusions in Cancer. http://cgapncinihgov/Chromosomes/Mitelman2018.

[33] Dastugue N., Lafage-Pochitaloff M., Pages M.P., Radford I., Bastard C., Talmant P., Mozziconacci M.J., Leonard C., Bilhou-Nabera C., Cabrol C. (2002). Cytogenetic profile of childhood and adult megakaryoblastic leukemia (M7): A study of the Groupe Francais de Cytogenetique Hematologique (GFCH). Blood.

[34] Alter B.P., Caruso J.P., Drachtman R.A., Uchida T., Velagaleti G.V., Elghetany M.T. (2000). Fanconi anemia: Myelodysplasia as a predictor of outcome. Cancer Genet. Cytogenet..

[35] Fenaux P., Lai J.L., Quiquandon I., Preudhomme C., Dupriez B., Facon T., Lorthois C., Lucidarme D., Bauters F. (1991). Therapy related myelodysplastic syndrome and leukemia with no “unfavourable” cytogenetic findings have a good response to intensive chemotherapy: A report on 15 cases. Leuk. Lymphoma.

[36] Borel C., Dastugue N., Cances-Lauwers V., Mozziconacci M.J., Prebet T., Vey N., Pigneux A., Lippert E., Visanica S., Legrand F. (2012). PICALM-MLLT10 acute myeloid leukemia: A French cohort of 18 patients. Leuk. Res..

[37] Babicka L., Ransdorfova S., Brezinova J., Zemanova Z., Sindelarova L., Siskova M., Maaloufova J., Cermak J., Michalova K. (2007). Analysis of complex chromosomal rearrangements in adult patients with MDS and AML by multicolor FISH. Leuk. Res..

[38] Lessard M., Helias C., Struski S., Perrusson N., Uettwiller F., Mozziconacci M.J., Lafage-Pochitaloff M., Dastugue N., Terre C., Brizard F. (2007). Fluorescence in situ hybridization analysis of 110 hematopoietic disorders with chromosome5 abnormalities: Do de novo and therapy-related myelodysplastic syndrome-acute myeloid leukemia actually differ?. Cancer Genet. Cytogenet..

[39] Lange K., Holm L., Vang Nielsen K., Hahn A., Hofmann W., Kreipe H., Schlegelberger B., Gohring G. (2010). Telomere shortening and chromosomal instability in myelodysplastic syndromes. Genes Chromosom. Cancer.

[40] Le Beau M.M., Albain K.S., Larson R.A., Vardiman J.W., Davis E.M., Blough R.R., Golomb H.M., Rowley J.D. (1986). Clinical and cytogenetic correlations in 63 patients with therapy-related myelodysplastic syndromes and acute nonlymphocytic leukemia: Further evidence for characteristic abnormalities of chromosomes no. 5 and 7. J. Clin. Oncol..

[41] Jeandidier E., Dastugue N., Mugneret F., Lafage-Pochitaloff M.M., Mozziconacci M.J., Herens C., Michaux L., Verellen-Dumoulin C., Talmant P., Cornillet-Lefebvre P. (2006). Abnormalities of the long arm of chromosome 21 in 107 patients with hematopoietic disorders: A collaborative retrospective study of the Groupe Francais de Cytogenetique Hematologique. Cancer Genet. Cytogenet..

[42] Yang Y., Xin X., Fu X., Xu D. (2018). Expression pattern of human SERPINE2 in a variety of human tumors. Oncol. Lett..

[43] De Pitta C., Tombolan L., Campo Dell’Orto M., Accordi B., te Kronnie G., Romualdi C., Vitulo N., Basso G., Lanfranchi G. (2005). A leukemia-enriched cDNA microarray platform identifies new transcripts with relevance to the biology of pediatric acute lymphoblastic leukemia. Haematologica.

[44] Dambruoso I., Boni M., Rossi M., Zappasodi P., Calvello C., Zappatore R., Cavigliano P.M., Giardini I., Rocca B., Caresana M. (2012). Detection of *TET2* abnormalities by fluorescence in situ hybridization in 41 patients with myelodysplastic syndrome. Cancer Genet..

[45] Soares-Ventura E.M., Mkrtchyan H., de Jesus Marques-Salles T., Silva M., Santos N., de Araujo Silva Amaral B., Liehr T., Abdelhay E., Silva M.L., Muniz M.T. (2011). Molecular cytogenetics reveals complex karyotype in apparent t(8;13) therapy-related acute myeloid leukemia M2 after fibrosarcoma. Leuk. Res..

[46] Walker A., Mrozek K., Kohlschmidt J., Rao K.W., Pettenati M.J., Sterling L.J., Marcucci G., Carroll A.J., Bloomfield C.D., Alliance for Clinical Trials in Oncology (2013). New recurrent balanced translocations in acute myeloid leukemia and myelodysplastic syndromes: Cancer and leukemia group B 8461. Genes Chromosom. Cancer.

[47] La Starza R., Crescenzi B., Nofrini V., Barba G., Matteucci C., Brandimarte L., Pierini V., Testoni N., Musto P., Paolini S. (2012). FISH analysis reveals frequent co-occurrence of 4q24/TET2 and 5q and/or 7q deletions. Leuk. Res..

[48] De Oliveira F.M., Miguel C.E., Lucena-Araujo A.R., de Lima A.S., Falcao R.P., Rego E.M. (2013). FISH analysis for *TET2* deletion in a cohort of 362 Brazilian myeloid malignancies: Correlation with karyotype abnormalities. Med. Oncol..

[49] Peniket A.J. (2005). Del(9q) acute myeloid leukaemia: Clinical and cytological characteristics and prognostic implications. Br. J. Haematol..

[50] Fonatsch C., Gudat H., Lengfelder E., Wandt H., Silling-Engelhardt G., Ludwig W.D., Thiel E., Freund M., Bodenstein H., Schwieder G. (1994). Correlation of cytogenetic findings with clinical features in 18 patients with inv(3)(q21q26) or t(3;3)(q21;q26). Leukemia.

[51] Lessard M., Struski S., Leymarie V., Flandrin G., Lafage-Pochitaloff M., Mozziconacci M.J., Talmant P., Bastard C., Charrin C., Baranger L. (2005). Cytogenetic study of 75 erythroleukemias. Cancer Genet. Cytogenet..

[52] Lai J.L., Zandecki M., Fenaux P., Le Baron F., Bauters F., Cosson A., Deminatti M. (1990). Translocations (5;17) and (7;17) in patients with de novo or therapy-related myelodysplastic syndromes or acute nonlymphocytic leukemia. A possible association with acquired pseudo-Pelger-Huet anomaly and small vacuolated granulocytes. Cancer Genet Cytogenet..

[53] Kuchinskaya E., Heyman M., Grander D., Linderholm M., Soderhall S., Zaritskey A., Nordgren A., Porwit-Macdonald A., Zueva E., Pawitan Y. (2005). Children and adults with acute lymphoblastic leukaemia have similar gene expression profiles. Eur. J. Haematol..

[54] Chen C.C., Yang C.F., Lee K.D., You J.Y., Yu Y.B., Ho C.H., Tzeng C.H., Chau W.K., Hsu H.C., Gau J.P. (2007). Complex karyotypes confer a poor survival in adult acute myeloid leukemia with unfavorable cytogenetic abnormalities. Cancer Genet. Cytogenet..

[55] Wyandt H.E., Chinnappan D., Ioannidou S., Salama M., O’Hara C. (1998). Fluorescence in situ hybridization to assess aneuploidy for chromosomes 7 and 8 in hematologic disorders. Cancer Genet. Cytogenet..

[56] Glenn L.D., Sanger W.G., Kessinger A., Vaughan W.P. (1988). Failure of karyotypic instability to predict clinical progression in patients with dysmyelopoietic syndromes. Hematol. Pathol..

[57] Viguie F., Aboura A., Bouscary D., Ramond S., Delmer A., Tachdjian G., Marie J.P., Casadevall N. (2005). Common 4q24 deletion in four cases of hematopoietic malignancy: Early stem cell involvement?. Leukemia.

[58] He Y.F., Li B.Z., Li Z., Liu P., Wang Y., Tang Q., Ding J., Jia Y., Chen Z., Li L. (2011). Tet-mediated formation of 5-carboxylcytosine and its excision by TDG in mammalian DNA. Science.

[59] Ito S., D’Alessio A.C., Taranova O.V., Hong K., Sowers L.C., Zhang Y. (2010). Role of Tet proteins in 5mC to 5hmC conversion, ES-cell self-renewal and inner cell mass specification. Nature.

[60] Figueroa M.E., Lugthart S., Li Y., Erpelinck-Verschueren C., Deng X., Christos P.J., Schifano E., Booth J., van Putten W., Skrabanek L. (2010). DNA methylation signatures identify biologically distinct subtypes in acute myeloid leukemia. Cancer Cell.

[61] Moran-Crusio K., Reavie L., Shih A., Abdel-Wahab O., Ndiaye-Lobry D., Lobry C., Figueroa M.E., Vasanthakumar A., Patel J., Zhao X. (2011). *Tet*2 loss leads to increased hematopoietic stem cell self-renewal and myeloid transformation. Cancer Cell.

[62] Quivoron C., Couronne L., Della Valle V., Lopez C.K., Plo I., Wagner-Ballon O., Do Cruzeiro M., Delhommeau F., Arnulf B., Stern M.H. (2011). TET2 inactivation results in pleiotropic hematopoietic abnormalities in mouse and is a recurrent event during human lymphomagenesis. Cancer Cell.

[63] Tefferi A., Pardanani A., Lim K.H., Abdel-Wahab O., Lasho T.L., Patel J., Gangat N., Finke C.M., Schwager S., Mullally A. (2009). TET2 mutations and their clinical correlates in polycythemia vera, essential thrombocythemia and myelofibrosis. Leukemia.

[64] Delhommeau F., Dupont S., Della Valle V., James C., Trannoy S., Masse A., Kosmider O., Le Couedic J.P., Robert F., Alberdi A. (2009). Mutation in TET2 in myeloid cancers. N. Engl. J. Med..

[65] Ko M., Huang Y., Jankowska A.M., Pape U.J., Tahiliani M., Bandukwala H.S., An J., Lamperti E.D., Koh K.P., Ganetzky R. (2010). Impaired hydroxylation of 5-methylcytosine in myeloid cancers with mutant TET2. Nature.

[66] Ko M., An J., Bandukwala H.S., Chavez L., Aijo T., Pastor W.A., Segal M.F., Li H., Koh K.P., Lahdesmaki H. (2013). Modulation of TET2 expression and 5-methylcytosine oxidation by the CXXC domain protein IDAX. Nature.

[67] Moon R.T., Kohn A.D., De Ferrari G.V., Kaykas A. (2004). WNT and beta-catenin signalling: Diseases and therapies. Nat. Rev. Genet..

[68] Barker N., Clevers H. (2006). Mining the Wnt pathway for cancer therapeutics. Nat. Rev. Drug Discov..

[69] Moon R.T., Bowerman B., Boutros M., Perrimon N. (2002). The promise and perils of Wnt signaling through beta-catenin. Science.

[70] Logan C.Y., Nusse R. (2004). The Wnt signaling pathway in development and disease. Annu. Rev. Cell Dev. Biol..

[71] Gelsi-Boyer V., Trouplin V., Adelaide J., Bonansea J., Cervera N., Carbuccia N., Lagarde A., Prebet T., Nezri M., Sainty D. (2009). Mutations of polycomb-associated gene ASXL1 in myelodysplastic syndromes and chronic myelomonocytic leukaemia. Br. J. Haematol..

[72] Kojima T., Shimazui T., Hinotsu S., Joraku A., Oikawa T., Kawai K., Horie R., Suzuki H., Nagashima R., Yoshikawa K. (2009). Decreased expression of CXXC4 promotes a malignant phenotype in renal cell carcinoma by activating Wnt signaling. Oncogene.

[73] Nguyen A.V., Albers C.G., Holcombe R.F. (2010). Differentiation of tubular and villous adenomas based on Wnt pathway-related gene expression profiles. Int. J. Mol. Med..

[74] Lu H., Sun J., Wang F., Feng L., Ma Y., Shen Q., Jiang Z., Sun X., Wang X., Jin H. (2013). Enhancer of zeste homolog 2 activates wnt signaling through downregulating CXXC finger protein 4. Cell Death & Disease.

[75] Chen E., Schneider R.K., Breyfogle L.J., Rosen E.A., Poveromo L., Elf S., Ko A., Brumme K., Levine R., Ebert B.L. (2015). Distinct effects of concomitant Jak2V617F expression and Tet2 loss in mice promote disease progression in myeloproliferative neoplasms. Blood.

[76] Kiladjian J.J., Masse A., Cassinat B., Mokrani H., Teyssandier I., le Couedic J.P., Cambier N., Almire C., Pronier E., Casadevall N. (2010). Clonal analysis of erythroid progenitors suggests that pegylated interferon alpha-2a treatment targets JAK2V617F clones without affecting TET2 mutant cells. Leukemia.

[77] Masarova L., Yin C.C., Cortes J.E., Konopleva M., Borthakur G., Newberry K.J., Kantarjian H.M., Bueso-Ramos C.E., Verstovsek S. (2017). Histomorphological responses after therapy with pegylated interferon alpha-2a in patients with essential thrombocythemia (ET) and polycythemia vera (PV). Exp Hematol. Oncol..

[78] Tefferi A., Sirhan S., Sun Y., Lasho T., Finke C.M., Weisberger J., Bale S., Compton J., LeDuc C.A., Pardanani A. (2009). Oligonucleotide array CGH studies in myeloproliferative neoplasms: Comparison with JAK2V617F mutational status and conventional chromosome analysis. Leuk. Res..

[79] Borze I., Mustjoki S., Juvonen E., Knuutila S. (2008). Oligoarray comparative genomic hybridization in polycythemia vera and essential thrombocythemia. Haematologica.

[80] Rumi E., Harutyunyan A., Elena C., Pietra D., Klampfl T., Bagienski K., Berg T., Casetti I., Pascutto C., Passamonti F. (2011). Identification of genomic aberrations associated with disease transformation by means of high-resolution SNP array analysis in patients with myeloproliferative neoplasm. Am. J. Hematol..

[81] Shaffer L.G., McGowan-Jordan J., Schmid M., ISCN (2013). An International System for Human Cytogenetic Nomenclature.

[82] Kjeldsen E., Roug A.S. (2012). A novel unbalanced de novo translocation der(5)t(4;5)(q26;q21.1) in adult T-cell precursor lymphoblastic leukemia. Mol. Cytogenet..

