# Supplementary material for: A Novel Acquired t(2;4)(q36.1;q24) with a Concurrent Submicroscopic del(4)(q23q24) in An Adult with Polycythemia Vera
Source: Cancers (Basel). 2018 Jun 25;10(7):214. doi: 10.3390/cancers10070214 (PMC6071118; doi:10.3390/cancers10070214)
Supplement: Supplementary file 1 [file cancers-10-00214-s001.pdf]

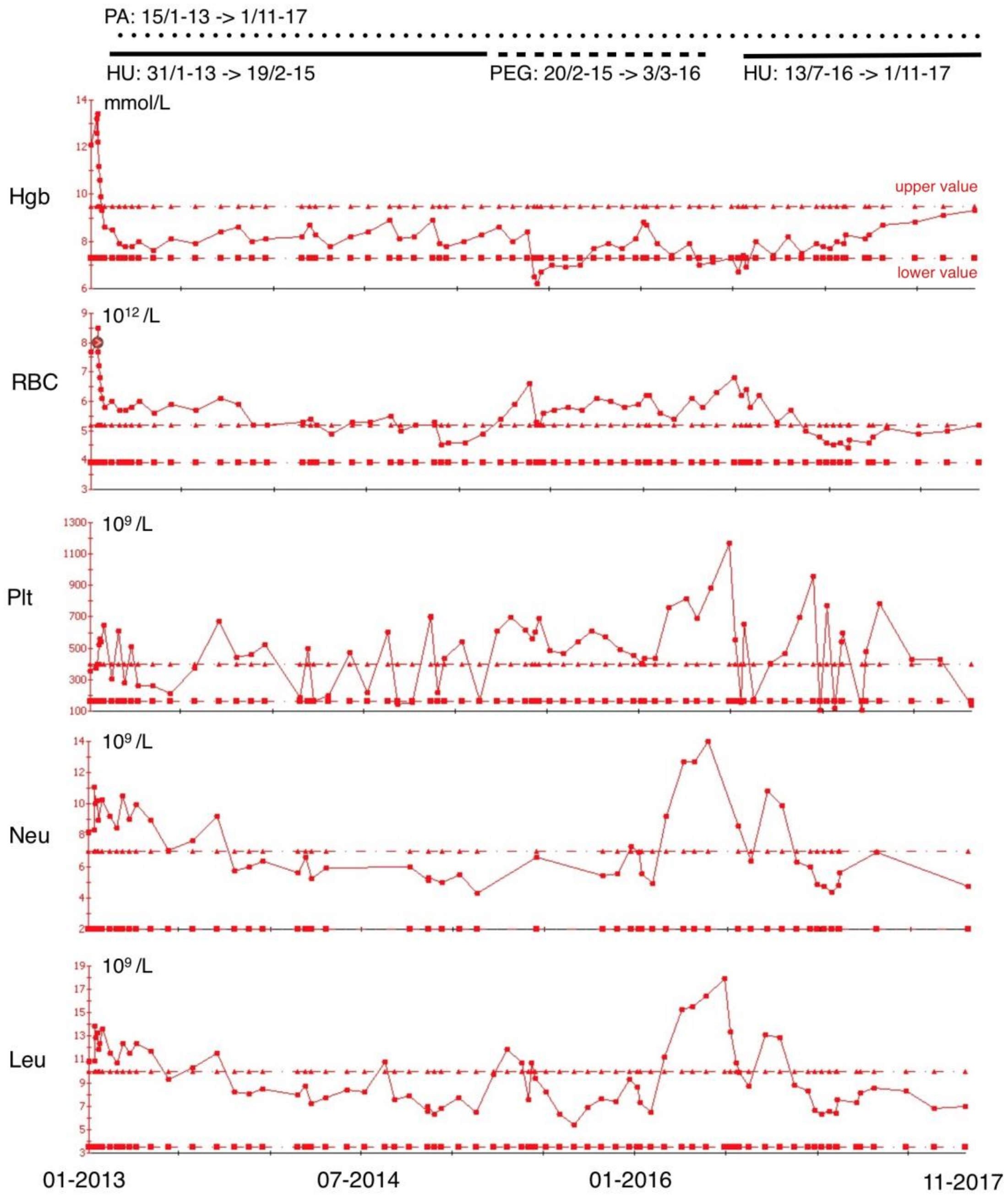

Supplemental Figure 1. Longitudinal evolution of biochemical parameters. Measured biochemical values of peripheral blood from the patient in the period from January 2013 (01-2013) at diagnosis of PV to follow-up in Novemer 2017 (11-2017) are depicted. Hemoglobin (Hgb); red blood cell count (RBC); platelet count (Plt); neutrophils count (Neu); and leucocyte count (Leu). Red horizontal lines in each panel indicate upper and lower reference values for each analysis. PA: phlebotomy and anti-thrombotic treatment; HU: hydroxy urea treatment; and PEG: pegylated interferon alfa-2a treatment.
